# On the multiple roles of the voltage gated sodium channel β1 subunit in genetic diseases

**DOI:** 10.3389/fphar.2015.00108

**Published:** 2015-05-18

**Authors:** Debora Baroni, Oscar Moran

**Affiliations:** Istituto di Biofisica – Consiglio Nazionale delle Ricerche, GenovaItaly

**Keywords:** voltage-gated sodium channel, channelopathies, epilepsy, cardiopathies, β1-subunit, GEFS+, Brugada syndrome

## Abstract

Voltage-gated sodium channels are intrinsic plasma membrane proteins that initiate the action potential in electrically excitable cells. They are composed of a pore-forming α-subunit and associated β-subunits. The β1-subunit was the first accessory subunit to be cloned. It can be important for controlling cell excitability and modulating multiple aspects of sodium channel physiology. Mutations of β1 are implicated in a wide variety of inherited pathologies, including epilepsy and cardiac conduction diseases. This review summarizes β1-subunit related channelopathies pointing out the current knowledge concerning their genetic background and their underlying molecular mechanisms.

## Introduction

Action potentials play a central role in most excitable cells, as neurons, skeletal and cardiac muscle, and endocrine cells. Action potential generation and propagation occur through, and are regulated by the function of voltage-gated sodium channels (NaCh), proteins with selective pores for sodium ions that span the cell membrane. In mammals, NaCh are heterotrimeric complexes composed of a pore-forming α-subunit (~260 kDa), a non-covalently associated β1- or β3-subunit and a covalently associated β2- or β4-subunit (Messner and Catterall, 1985; Catterall, 2012).

There are nine NaChs α-subunit pore-forming isoforms encoded by different genes, termed Nav1.1 to Nav1.9, and an atypical non-voltage-dependent one, named NavX (Catterall, 2012). In humans, Nav1.1, Nav1.2, Nav1.3, and Nav1.6 are abundantly expressed in the central nervous system (CNS) and in the peripheral nervous system (PNS); Nav1.1 and Nav1.6 are also expressed in adult ventricular myocytes. Nav1.4 is abundant in adult skeletal muscle while Nav1.5 is expressed predominantly in heart. Nav1.7, Nav1.8, and Nav1.9 are preponderantly located in the PNS (**Table 1**). The α-subunit isoforms show a high degree of amino-acid sequence identity. Vertebrate α-subunits contain four homologous but non-identical domains (I–IV), each of which contains six transmembrane segments (S1–S6). The residues between S5 and S6 form the channel pore (P-loop) and control ion selectivity and permeation. Positively charged S4 segments act as voltage sensors.

**Table 1:** Types of human sodium channels (NaCh) α and β subunits and their tissue distribution.

Gene	Chromosome	Protein	Uniprot code*	Tissue expression
**α-subunits**
*SCN1A*	2q24.3	Nav1.1 or α1.1	P35498	Cell bodies of central neurons, T-tubules in myocytes axon initial segments
*SCN2A*	2q24.3	Nav1.2 or α1.2	Q99250	Central neurons, mainly localizated in unmyelinated and premyelinated axons
*SCN3A*	2q24.3	Nav1.3 or α1.3	Q9NY46	Cell bodies of central neurons, cardiac myocytes
*SCN4A*	17q23.3	Nav1.4 or α1.4	P35499	Skeletal muscles
*SCN5A*	3p21-22	Nav1.5 or α1.5	Q86V90	Cardiac myocytes, immature and denervated skeletal muscles, certain brain neurons
*SCN8A*	12q13	Nav1.6 or α1.6	Q9UQD0	Somatodendritic distribution in output neurons of cerebellum, cerebral cortex, hippocampus; Purkinje cells in cerebellar granule cell layer, astrocytes, Schwann cells, axon initial segments, dorsal root ganglia, nodes of Ranvier in peripheral and central nervous systems, T-tubules in cardiac myocytes
*SCN9A*	2q24	Nav1.7 or α1.6	Q15858	Dorsal root ganglia neurons, sympathetic neurons, Schwann cells, neuroendocrine cells
*SCN10A*	3p22.2	Nav1.8 or α1.8	Q9Y5Y9	Dorsal root ganglia neurons, human heart, intracardiac neurons
*SCN11A*	3p22.2	Nav1.9 or α1.9	Q9UI33	C-type neurons in dorsal root ganglia
*SCN7A*	2q24.3	NavX	Q01118	Dorsal root ganglia neurons, hippocampus, thalamus, cerebellum, median preoptic nucleus, circumventricular organs, Peripheral nervous system (PNS), heart, skeletal muscle, uterus


**β -subunits**
*SCN1B*	19q13.1	SCN1b or β1	Q07699	Ubiquitous: central and peripheral neurons, glia, skeletal and cardiac muscles
*SCN1B*	19q13.1	SCN1bB or β1B		Cortical neurons, Cerebellar Purkinje cells, Deep cerebellar nuclei, Ventral horn neurons, Dorsal root ganglia neurons, peripheral nerves
*SCN2B*	11q23	SCN2b or β2	Q5U0K8	Central and peripheral neurons, glia, cardiac muscles
*SCN3B*	11q23.3	SCN3Bor β3	Q9NY72	Central and peripheral neurons, adrenal glands, kidney
*SCN4B*	11q23.3	SCN4b or β4	Q8IWT1	Central and peripheral neurons, glia, skeletal and cardiac muscles

Modified from Patiño et al. (2009) and Catterall (2012).

*http://www.uniprot.org

To date, five β-subunits have been identified in mammals: β1, its alternative splice variant β1B (previously called β1A), β2, β3, and β4. Each β-subunit is encoded by one of four genes, *SCN1B*–*SCN4B*. As well as α-subunits, β-subunits are highly expressed in excitable cells, including central and peripheral neurons, skeletal and cardiac muscle cells. They are also expressed in non-excitable cells such as astrocytes, radial glia, and Bergmann glia (**Table 1**).

β-subunits (~30–40 kDa) are single pass molecules with an extracellular N-terminus, a transmembrane-spanning segment, and an intracellular C-terminus. The β1B-subunit arising from the retention of a segment of intron 3 (exon 3A) does not include neither the transmembrane nor the intracellular domains, being a soluble protein (Qin et al., 2003; Patiño et al., 2009).

β1- and β3-subunits, which share 57% sequence homology, associate non-covalently with the α-subunits (Isom et al., 1992; McCormick et al., 1998; Morgan et al., 2000; Meadows et al., 2001; Spampanato et al., 2004), whereas β2- and β4-subunits, which have a high sequence homology, are covalently bound to the α-subunits by a disulphide bond (McCormick et al., 1998; Yu et al., 2003; Spampanato et al., 2004). All five β-subunits contain an extracellular immunoglobulin-like (Ig) domain homologous to V-type Ig-loop motif present in the Ig superfamily of cell adhesion molecules (CAMs), with a noteworthy homology to the CAM myelin P_0_ glycoprotein (Isom and Catterall, 1996; Morgan et al., 2000; Yu et al., 2003), a structural feature that enables them to function as CAMs (Isom et al., 1995a; Brackenbury and Isom, 2011; Calhoun and Isom, 2014). This protein motif has been confirmed in the crystallographic studies of the extracellular domain of the β3- and β4-subunits (Gilchrist et al., 2013; Namadurai et al., 2014).

Many studies have tried to demonstrate that β-subunits are able to fine-tune gating and kinetics of α-subunits expressed heterologously. There is no doubt that the classical roles of β-subunits as “conducing” modulators of Na^+^ current is of paramount importance in regulating ion flux and cell excitability. However, there is a clear trend in literature that underlines the importance of β-subunit “non-conducing” functions, including NaCh cell surface expression regulation, migration and pathfinding, cell adhesion and putative transcriptional modulation (Davis et al., 2004; Brackenbury et al., 2008, 2010; Baroni et al., 2013, 2014; Baroni and Moran, 2015). Furthermore, β-subunits are key players in a variety of pathologies, including epilepsy, cardiac arrhythmia, neuropsychiatric disorders, neuropathic and inflammatory pain, and cancer (Brackenbury and Isom, 2011). Thus, the understanding of the interactions between NaCh α- and β-subunits is of predominant importance, also in view of the exploitation of their therapeutic potential.

In this review we will focus on the multiple roles played by the β1-subunit, which has been the first ancillary subunit to be cloned and to be associated to human diseases. We will describe its mutations and illustrate some hypotheses formulated to attempt the explanation of the mechanisms that lead to β1 mutation-related pathologies.

## β1 Functions

From its molecular identification (Isom et al., 1992), the β1-subunit has been proposed to modulate gating and kinetics properties of NaCh, especially inactivation. Co-expression of rat β1-subunit with skeletal muscle or brain rat α-subunits in *Xenopus* oocytes has been proposed to increase the amplitude of the peak sodium current, accelerate inactivation, and shift the voltage-dependence of inactivation to more negative membrane potentials (Isom et al., 1992; Patton et al., 1994; Moran and Conti, 2001). However, data regarding the heterologous expression of β1 in mammalian cells are contradictory and different results have been described by different groups. It was reported that, in mammalian cells, the β1-subunit is able: to shift the inactivation curve to positive, negative or to not change the potential, to shift the activation curve to negative potentials or to not change it, to hasten the recovery from inactivation or to not change it, to increase or do not modify the density of sodium currents (Bendahhou et al., 1995; Isom et al., 1995b; Hayward et al., 1996; An et al., 1998; Kazen-Gillespie et al., 2000; Tammaro et al., 2002; Moran et al., 2003). It has also been proposed that β1-subunit modulates NaCh gating through the screening of the membrane surface charge (Johnson et al., 2004; Ferrera and Moran, 2006).

Beside the regulation of NaCh gating, it has been proposed that β1-subunit participates in cell–cell and cell–matrix adhesion, contributing to cellular aggregation, ankyrin recruitment, and neurite outgrowth (Srinivasan et al., 1998; Malhotra et al., 2000, 2002; Kazarinova-Noyes et al., 2001; Ratcliffe et al., 2001; Davis et al., 2004; McEwen et al., 2004). Finally, it was demonstrated that in excitable cells the β1-protein acts as a crucial element in the assembly and cell surface expression of the heteromeric complex of the sodium channel, determining the type and the amount of α-subunit to be expressed (Patiño et al., 2009). Indeed, over-expression and silencing of the NaCh accessory subunit, demonstrate that the β1 is able to regulate the NaCh expression, and it is also a key factor in the processes that determine which α-subunit is going to be expressed (Baroni et al., 2013, 2014).

Consistently with these properties, β1-subunit was demonstrated able to rescue trafficking-deficient Nav1.1 channels to the cell surface, thus influencing the disease severity caused by the lack of a properly functional NaCh α-subunit (Rusconi et al., 2007, 2009; Sugiura et al., 2012; Thompson et al., 2012; Bechi et al., 2015). Also in this case, disease severity may be severely influenced by the total or partial lack of the β1-subunit capability to traffic mutant Nav1.1 to the cell surface.

## β1-Linked Diseases

One of the most remarkable findings of research on the molecular properties of NaCh β1-subunit was the discovery that its mutations cause inherited diseases that selectively affect the CNS or the heart (Wallace et al., 1998; Antzelevitch, 2003; Fish and Antzelevitch, 2003; Watanabe et al., 2008; Escayg and Goldin, 2010). Unfortunately, the comprehension of the molecular mechanisms underlying the *SCN1B* mutation physiopathology is limited by the lack of a unique and exhaustive elucidation of the role played by this protein on the regulation of the NaCh. Evidences collected up to now suggest a model in which gene dosage may determine the severity of disease (Moran and Conti, 2001). For example, for *SCN1B* mutations related to CNS diseases, a single mutant allele may result in the development of a milder disease like generalized epilepsy with febrile seizures plus. In contrast, expression of two non-functional *SCN1B* alleles may result a more severe epileptic disease like the Dravet Syndrome.

**FIGURE 1:**
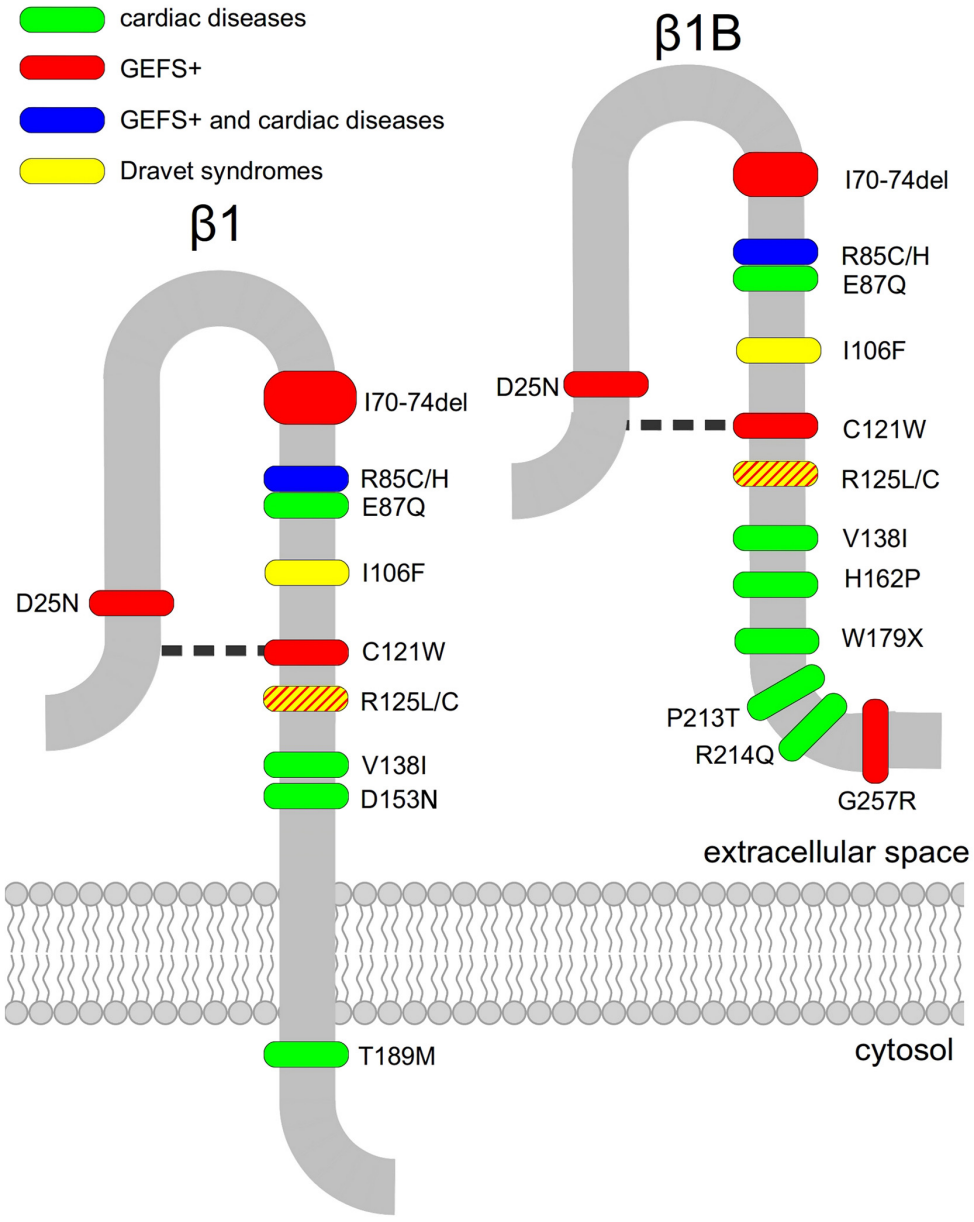
**Disease-linked mutations of voltage-gated sodium channel β1- and β1B-subunits**. Mutated residues associated with epilepsy (green), cardiac diseases (red) or both (blue) are marked. The disulphide bridge involving residue C121 is indicated as a broken line.

Another peculiarity that distinguishes *SCN1B* mutations linked either to CNS or to cardiac diseases is that, with the exception of the recently identified mutation G257R (Patiño et al., 2009) which is located in the β1B retained intronic region, all generalized epilepsy with febrile seizures plus (GEFS+) causing mutations are localized in the Ig-loop region (**Figure 1**), suggesting that the cell adhesion functions mediated by this region are clinically relevant (Brackenbury and Isom, 2011).

The inherited diseases caused by mutations in the NaCh β1-subunit described so far are:

### 1. Generalized Epilepsy with Febrile Seizures Plus (GEFS+)

Some mutations in *SCN1B* are linked to GEFS+ (OMIM:604233), an autosomal dominant inherited epilepsy. The first *SCN1B* mutation identified in GEFS+ was C121W (Wallace et al., 1998, 2002), caused by a 387C-to-G transversion in *SCN1B* gene. As a consequence, a key disulphide bond involved in maintaining the extracellular Ig-like loop is disrupted (Barbieri et al., 2012).

Functional studies of mutant rat C121W β1-subunit co-expressed with either with brain 1.2 or muscle 1.4 rat α-subunits in *Xenopus laevis* oocytes showed that the mutated β1-subunit loses its ability to modulate the acceleration of the inactivation rate of the sodium channel compared with wild type (WT) β1-subunit (Wallace et al., 1998; Moran and Conti, 2001). Interestingly, C121W-β1 heterologous expression in mammalian cells yielded contradictory results, depending on the α-subunit co-expressed and on the expression system. When co-expressed with human Nav1.3, human C121W-β1 causes a rightward shift of inactivation compared to the WT-β1, potentially increasing channel excitability (Meadows et al., 2002). On the contrary, when rat C121W-β1 is co-expressed with rat skeletal muscle Nav1.4, sodium channels recover more slowly from fast inactivation (Tammaro et al., 2002).

It has been argued that C121W-β1 acts as a dominant-negative subunit, competing with the WT-β1-subunit in the regulation of the NaCh α-subunit expression and activity (Moran and Conti, 2001; Meadows et al., 2002). In rat neuronal-like cells, the regulatory effect of the over-expression of rat β1-subunit on the α-subunit mRNA, protein and Na^+^ current levels is abolished by the epileptogenic C121W-β1; conversely, in rat cardiac cells mutation C121W does not alter the β1-subunit modulation of NaCh (Baroni et al., 2013). These findings demonstrate the tissue-specificity of the modulation of NaCh expression.

Successively, six other mutations, I70_E74del, R85C, R85H, G257R, R125L, and D25N were associated to GEFS+, R85C, and R85H are missense mutations of an evolutionary conserved arginine residue in the Ig-loop (Scheffer et al., 2007). When co-expressed with human Nav1.2, human R85H-β1 appeared to modulate the voltage-dependence of NaCh slow inactivation without any effect on other electrophysiological parameters, while co-expression of human Nav1.2 with human R85C-β1 had no detectable effects on any channel property, suggesting a complete loss of function mutant (Xu et al., 2007). Immunohistochemical studies on cells transiently transfected with β1 mutants R85C and R85H failed to detect them at the cell surface, indicating that they are trafficking defective (Xu et al., 2007). Conversely, in surface biotinylation assay, similarly to WT-β1, human β1-R85H was detected at the cell surface of stably transfected Chinese hamster lung 1610 cells (Patiño et al., 2011), pointing out the need for further investigations on the cellular localization of this mutant.

Unlike the other GEFS+- associated *SCN1B* mutations, that are located in the Ig-domain, the missense mutation G257R is located in the β1B retained intronic region (Patiño et al., 2011). Surface biotinylation assay revealed that differently from Chinese hamster lung 1610 cells stably transfected with human WT-β1, Chinese hamster lung 1610 cells permanently transfected with human G257R fail to show the mutant β1 at the plasma membrane (Patiño et al., 2011).

Mutation I70_E74del is a A-to-C transversion in the splice acceptor site of exon 3 of *SCN1B* gene, resulting in a deletion of five amino acids within the extracellular Ig-fold (Audenaert et al., 2003). Unfortunately, no functional data are available for this mutation. R125L is a GEFS+-associated mutation, caused by a 374G-to-T transversion in exon 3 of *SCN1B* gene. It determines the substitution of a highly conserved arginine in the extracellular domain of the protein (Fendri-Kriaa et al., 2011). Even though functional studies on this mutation are still not available, it can be hypothesized that mutation R125L causes electrostatic changes and a loss of hydrogen bonding in the Ig-loop region affecting the structure and stability of the protein. The last GEFS+-associated *SCN1B* mutation is D25N. This missense mutation is due to a 73G-to-A transversion in exon 2 of *SCN1B* and causes the neutralization of an charged residue in the Ig-loop (Orrico et al., 2009).

### 2. Dravet Syndrome (DS)

Dravet syndrome or severe myoclonic epilepsy of infancy (OMIM:607208) is a severe form of generalized epilepsy with febrile seizures, characterized by generalized tonic, clonic, and tonic–clonic seizures triggered at first by fever, arising shortly after birth. Cognitive development is normal until ~2 years of age, when it slows or stagnates (Dravet, 1978; Wolff et al., 2006). Classically, DS is considered to be a *SCN1A*-linked disease (Oguni et al., 2005; Korff and Nordli, 2006). However, a small but growing number of DS patients affected by mutations in *SCN1B* has been described. Differently from GEFS+, all the SCN1B mutations causing DS have been found in homozygosis.

The first *SCN1B* mutation identified in DS is R125C, which prevents normal trafficking of β1 to the cell surface and thus results in a functional null phenotype (Patiño et al., 2009). Chinese hamster lung 1610 cells stably transfected with the rat Nav1.2 subunit as well HEK cells permanently transfected with human Nav1.1 were further stably transfected with human WT- or R125C-β1. Western-blot analysis of cell fractions unequivocally demonstrated that human R125C is inefficiently expressed at the cell surface at physiological temperatures, but the overcome of this trafficking defect at a lower temperature permits the mutant β1-subunit to be fully capable of modulating sodium current (Patiño et al., 2009). Another *SCN1B* mutation linked to DS, I106F, is caused by a 316A>T nucleotide change resulting in residue substitution in the Ig-loop (Ogiwara et al., 2012). No functional data are available for this mutant protein.

Mouse models support the link between *SCN1B* and epilepsy. Scn1b-null mice have frequent spontaneous generalized seizures, display aberrant neuronal excitability, and have defects in neuronal development (Chen et al., 2004; Brackenbury et al., 2013). Importantly, abnormalities in brain development are observed at P5, prior to seizure onset, suggesting that structural alterations, aberrant cell adhesive interactions, and abnormal excitability early in development may be causative factors in epileptogenesis (Brackenbury et al., 2013). A knock-in mouse model of C121W-mediated GEFS+ displays hyper-excitability in specific sub-populations of central neurons, reduced dendritic arborisation of subicular pyramidal neurons, and increased susceptibility to febrile seizures (Wimmer et al., 2010; Hatch et al., 2014).

### 3. Brugada Syndrome (BrS)

Brugada syndrome (BrS) is a condition characterized by a distinct ST-segment elevation in the right precordial leads of the electrocardiogram and by an increased risk of cardiac arrhythmia and sudden death (Brugada and Brugada, 1992). The condition predominantly exhibits an autosomal dominant pattern of inheritance and incomplete penetrance. It has an average prevalence of 5:10000 worldwide, and is much more common in men than in women (Priori et al., 2002; Smits et al., 2002; Antzelevitch, 2003). The mean age of BrS clinical debut is 40 years; however, the first occurrence of symptoms may occur in early childhood or old age (Antzelevitch, 2003). Currently, BrS is associated to more than 100 mutations in seven genes (*SCN5A, GPD1L, CACNA1C, CACNB2, KCNE3, SCN3B*), including *SCN1B*.

E87Q is the first β1 mutation linked to BrS (BrS5, OMIM:612838), caused by a 259G-C transversion in exon 3 of the human *SCN1B* gene. It results in a substitution of the neutralization of a highly conserved glutamic acid within the Ig-loop, which is common to both the β1 and β1B transcripts. Functional studies of the E87Q mutation in transiently transfected CHO cells show that the co-expression of mutant human β1 or β1B with human cardiac Nav1.5 neither increases the sodium current nor produces a negative shift in the voltage dependence of the activation curve with respect to cells transfected with WT-β1 or -β1B and Nav 1.5. Mutant E87Q-β1 or -β1B shifted only the voltage dependence of inactivation to negative potentials (Watanabe et al., 2008).

Another mutation linked to BrS is W179X that has been found in β1B. It is a non-sense mutation caused by a 536G-A transition in exon 3A of the *SCN1B* gene (Watanabe et al., 2008). A variant of this mutation, produced by a 537G-A transition, also causes the W179X mutation. This variant has been correlated with cardiac conduction defects without any BrS symptom (Watanabe et al., 2008). It is conceivable that the lack of a β1 protein causes a disease by simply haploinsufficiency. Functional studies of W179X mutation showed that the co-expression of human W179X-β1B with human cardiac Nav1.5 failed to increase sodium currents and did not modulate the activation and inactivation (Watanabe et al., 2008).

The BrS linked-mutation, R214Q, has been found in exon 3A of β1B-subunit (Hu et al., 2012) It is due to a 641G-to-A transversion. Sodium currents of cells transfected with human *SCN5A* and *SCN1Bb*-R214Q resulted 56.5% smaller than that of *SCN5A* plus *SCN1Bb*-WT and 33.05% smaller than that of cells transfected with the sole *SCN5A*. Furthermore, R214Q caused no significant shift in steady-state inactivation and activation, but slowed recovery from inactivation (Hu et al., 2012).

SCN1B BrS-linked H162P mutation was found in a Danish patient by Holst et al. (2012). As the patient did not completely fulfill the diagnostic criteria for BrS and no functional data are available, further investigations would be mandatory to confirm the clinical relevance of this mutant.

Finally two *SCN1B* mutations, V138I and T189M, have been related to sudden unexplained nocturnal death syndrome (SUNDS), a disorder whose electrocardiogram (ECG) characteristics and clinical phenotype are very similar to BrS (Liu et al., 2014).

### 4. Atrial Arrhythmias

R85H and D153N are *SCN1B* mutations that have been associated with familiar atrial fibrillation (ATFB13 OMIM:615377). R85H is located in the Ig-loop, and thus affects both β1 and β1B. Conversely, D153N is located in exon 4 of *SCN1B* and thus can only affect β1. Both mutations result in a reduction of sodium currents in heterologous expression systems. In comparison with human WT-β1 co-expressed with human SCN5A in CHO cells, D153N does not affect the sodium channel activation or inactivation. However, R85H resulted in a positive shift of voltage-dependence of both, activation and inactivation (Watanabe et al., 2009).

R85H, has been also reported as an epilepsy mutation in patients from two families without history of seizure disorders (Scheffer et al., 2007). Further functional studies would be mandatory to confirm the clinical relevance of this mutant, that represents an exception among *SCN1B* mutations. In fact all the *SCN1B* mutations identified so far have demonstrated to selectively affect the CNS or the heart.

### 5. Long QT- Syndrome (LQTS)

Long QT- syndrome (LQTS) is a cardiovascular disorder associated with syncopal episodes, torsades de pointes, ventricular fibrillation, and sudden death. This syndrome is characterized by prolonged QT-interval in the ECG because of an abnormality in cardiac repolarization. At least 15 forms of LQTS have been identified, each with specific associated genes, variations in penetrance, allele dominance, and co-morbidities.

A recent report identified mutation P213T of β1B to cause LQTS (OMIM: 611819; Giudicessi and Ackerman, 2013). When heterologously co-expresed in HEK cells, both human β1B-WT and β1B-P213T increased sodium currents with respect to expression of human Nav1.5 alone. The activation voltage dependence curve was significantly shifted to the left in cells co-expressing Nav1.5 and β1B-P213T compared with Nav1.5 β1B-WT, while the inactivation voltage dependence curve was not affected by the mutation. P213T of β1B significantly accelerates the recovery from inactivation. Furthermore, the probability of having more channels in the slow inactivated state resulted significantly lower for Nav1.5β1B-P213T than for Nav1.5 β1B-WT. This change could lead to higher channel availability, unbalancing the currents that determine the duration of action potentials, and determining the condition for LQTS onset (Riuró et al., 2014).

Evidence in transgenic mice suggests that β1 subunit is involved in normal cardiac function and that mutations of SCN1B can result in disease. Consistent with LQTS, Scn1b-null mice have abnormal cardiac action potentials evidenced by prolonged QT intervals that persist after pharmacological autonomic blockade (Lopez-Santiago et al., 2007). Scn1b-null ventricular myocytes also display increased peak and persistent Na^+^ current relative to WT cells (Lopez-Santiago et al., 2007).

## Concluding Remarks

A growing list of *SCN1B* mutations linked to inherited diseases reveals the important roles that the β1-subunit plays in the NaCh-function. β1-subunit channelopathies belong to two categories: epileptic syndromes and cardiac arrhythmias. Each *SCN1B* mutation seems to have a tissue-selectivity whose molecular mechanism is far to be elucidated. Another peculiarity is the wide spectrum of phenotypes and clinical manifestations that can be observed in patients affected by the same *SCN1B* mutation.

The comprehension of the pathophysiology of diseases caused by mutations of β1-subunit is severely limited by the understanding of the functional role of the β1-subunit. The β1-subunit was recognized as a part of the NaCh complex since the early attempts to identify the molecules that compose this channel. However, even though the molecular identification of the β1-subunit, and the possibility to express it in heterologous systems, the role of this protein is still controversial. The β1-subunit could play three different roles. It has been claimed that β1-subunit is involved in the fine tuning of the NaCh gating. This proposal comes from the heterologous expression of β1-subunit in *Xenopus* oocytes, where it dramatically regulates the NaCh inactivation. However, when the β1-subunit is heterologously expressed in mammalian cells results are contradictory, and, in general, its role seems to be correlated to an indirect effect by charge surface modifications and not to a specific NaCh modulation. The second possible role of the β1-subunit is associated to the interactions of the NaCh with the cytoskeleton and the extracellular matrix, determining the correct docking of the NaCh in specific regions of the plasma membrane. A further role in modulating the gene expression, and therefore the amount and quality of NaCh α-subunits has been also recently illustrated. All three possible roles could be implicated in the genesis of the diseases caused by β1-subunit mutations but none of these hypotheses has been incontrovertibly demonstrated yet. As occurs with any biological mechanism with a high degree of complexity, one could hypothesize that other genes – for example, genes encoding some of the β1-subunit interacting proteins, may likely exert their influence on the severity of the diseases linked to β1-subunit mutations or determine the tissue-specificity. The disclosure of this specific genetic relationships will not only shed new light on the biology of NaCh heteromeric complex but also provide critical information to design more appropriate pharmacological therapies.

## Conflict of Interest Statement

The authors declare that the research was conducted in the absence of any commercial or financial relationships that could be construed as a potential conflict of interest.
